# Mean Fluoride Concentration in Drinking Water Sources of a Municipality: A Descriptive Cross-sectional Study

**DOI:** 10.31729/jnma.7898

**Published:** 2022-11-30

**Authors:** Rajib Chaulagain, Ayam Chhatkuli, Ashim Raj Shrestha, Bikram Karki Chhetri, Sadhana Pandey

**Affiliations:** 1 Department of Oral Pathology, Chitwan Medical College, Bharatpur, Chitwan, Nepal; 2 Nidan Dental Care, Tamghas, Gulmi, Nepal; 3 Gulmi Hospital, Tamghas, Gulmi, Nepal; 4 Health Foundation Nepal, Ghorahi, Dang, Nepal

**Keywords:** *dental caries*, *drinking water*, *fluoride*

## Abstract

**Introduction:**

Fluoride is essential for the growth and development of teeth and bone. Excess or less fluoride consumption can have harmful effects on our bodies. Fluoride level of 0.5-1.5 mg/l is said to be optimized according to the World Health Organization. The level of fluoride varies among the different geographical regions and water sources. It is essential to find out the fluoride content of various water sources used for drinking purposes. The aim of this study was to find the mean concentration of fluoride in drinking water sources of a municipality.

**Methods:**

A descriptive cross-sectional study was conducted in a Municipality. The study was conducted from 1 December 2021 to 30 December 2021 after receiving ethical approval from the Ethical Review Board (Reference number: 1134). Water samples were collected and the fluoride content was estimated using 2-parasulfophenylazo-1,8-dihydroxy-3,6-napthalene-disulfonate colorimetric method. The data collected from the laboratory were calculated and presented in the form of a table. Point estimate and 95% Confidence Interval were calculated.

**Results:**

The mean value of fluoride content in 160 collected water samples was 0.369±0.275 mg/l (0.33-0.41, 95% Confidence Interval). Among the different wards, the fluoride content was 0.708±0.27 mg/l in ward number 12 followed by a fluoride content of 0.57±0.19 mg/l in ward number 5.

**Conclusions:**

In this study, the mean fluoride levels were lower when compared with similar studies conducted in similar settings. The levels were lower than that recommended by the World Health Organization. The various controlled methods of fluoridation have to be quickly initiated. Other means of fluoride consumption, like the use of fluoridated toothpaste, has to be recommended.

## INTRODUCTION

Fluoride, an elemental form of Fluorine, is the 13^th^ most abundant element present in the earth's crust. It is naturally present in water, air and soil.^1^ However, its distribution is unequal in nature,^2,3^ and is affected by the geochemical settings, chemical characteristics of groundwater, pH and climate.^4^

Fluoride should always be consumed at an optimum level to avoid its deleterious effects.^5-8^ Excess fluoride has been shown to be associated with bone fracture.^9^ It may also cause decay of teeth,^10^ and damage to kidneys, nerves and muscles.^6,9^ For this reason, World

Health Organization (WHO) has set the optimum level of fluoride at 0.5-1.5 mg/l in drinking water.^6^ In Nepal, published studies have shown less fluoride content in the drinking water supply in Nepal.^11-13^ Such a study has not been done in the Western part of Nepal.

The aim of this study was to measure the mean concentration of fluoride in the drinking water of a municipality.

## METHODS

A descriptive cross-sectional study was designed and conducted to measure the fluoride concentration in the drinking water of Resunga Municipality, Tamghas, Gulmi. This Municipality at present has 14 wards. The data was collected from 1 December 2021 to 30 December 2021. Ethical approval was obtained from Nepal Health Research Council Ethical Review Board (Reference number: 1134) while prior permission from the Office of the Municipal Executive, Resunga Municipality (Reference number: 1134) was also obtained. The inclusion criteria were water commonly used for drinking and packaged water bottles while the exclusion criteria were regions difficult to reach and contaminated water sources. The sample size was calculated using formula:


$$\begin{array}{ll} \text{n} & ={\text{Z}}^{2}\times \frac{\sigma }{{\text{e}}^{\text{2}}}\\ & =1.96^{2}\times \frac{0.32^{2}}{0.05^{2}}\\ & =158 \end{array}$$

Where,

n= minimum required sample size
Z= 1.96 at 95% Confidence Interval (CI)
σ = standard deviation, 0.32
e= margin of error, 0.05

The calculated sample was 158. However, in the study, a total of 160 samples were included. In this study, a consecutive sampling technique was utilised to collect the water samples to attain the desired sample size.

Water from the different sources was collected in good quality 200 ml sterilised polythene bottles. Each bottle was properly labelled with the site and date of data collection. No preservatives and chemicals were added to the bottles. The collected samples were then placed at room temperature and then transported to Nepal Batawaraniya Sewa Kendra (ISO-certified laboratory), Biratnagar. The fluoride measurement was done by 2-parasulfophenylazo-1,8-dihydroxy- 3,6-napthalene-disulfonate (SPADNS) Colorimetric method which deals with the dissociation reaction between the Fluoride and Zirconium dye lake where the product ultimately formed is a colourless anion. The increase in the amount of Fluoride was associated with a lighter colour, while decrease in the amount of Fluoride showed darker colour. The resultant colour intensity was measured at 570 nm.

The fluoride level measured from the laboratory of each sample was transferred to the Microsoft Excel file according to the respective codes and then exported to IBM SPSS version 20.0. Point estimate and 95% Confidence Interval were calculated.

## RESULTS

The mean value of fluoride content in collected 160 water samples was 0.369±0.275 mg/l (0.33-0.41, 95% CI). It was observed in ward number 12 the fluoride content was 0.708±0.27 mg/l followed by ward number 5 with a fluoride content of 0.57±0.19 mg/l (Table 1).

**Table 1:** Fluoride concentration in different wards of Resunga municipality (n= 160).

Ward number	Number of samples	Mean±SD
1	10	0.4610±0.15
2	10	0.5320±0.24
3	7	0.3629±0.19
4	18	0.2061±0.07
5	14	0.5721±0.19
6	11	0.4318±0.23
7	3	0.2167±0.16
8	34	0.1921±0.29
9	5	0.5620±0.33
10	16	0.3794±0.20
11	8	0.1713±0.08
12	13	0.7085±0.27
13	8	0.4000±0.27
14	3	0.0900±0.07

## DISCUSSION

Fluoride is one of the most ubiquitous elements present in the earth crust.^14^ It is the most electronegative and reactive element.^4,6^ Literature have reported that surface water has low fluoride content whereas depending upon the different geographical conditions the groundwater can vary in Fluoride content.^14-16^ Groundwater with arid climatic conditions can also have an impact on the fluoride content.^4,17^ Fluorides are also termed a double-edged sword. The lesser or excess consumption can have toxic effects on health.^5,6^ Excess amounts of fluoride has also a detrimental effect on our oral cavity especially teeth where dental fluorosis occurs and in the extreme case leads to skeletal fluorosis.^14,18,19^ People mainly intake fluoride in their bodies through drinking water and from different sources.^1^ However, in different geographical settings, the levels of fluoride in the different sources are also different. Due to this, the effects of fluoride may also vary among the regions. For this WHO has recommended a guideline of optimum level of 1.5 mg/l of fluoride. WHO has also recommended in their guidelines that artificial water fluoridation is 0.5-1 mg/l.^20^ In this study, the investigators aimed to estimate mean concentration of fluoride in the drinking water of Resunga municipality. There are different methods available to measure the concentration of fluoride in drinking water such as ion chromatography, colourimetric, and spectrophotometric methods.^21^ In this study, we used SPADNS colorimetric method, which is simple and quick.

The present study confirmed that the fluoride level in the Resunga municipality was below the optimum level. Except for a few wards, most of the wards had below optimum levels of fluoride. However, four wards 2, 5, 9, and 12 showed fluoride levels within the range recommended by WHO, but at a lower level. In line with the present study findings, other studies in Nepal also showed similar results. A study collected water samples from eight water distribution schemes in Kathmandu valley and showed that the fluoride level in the drinking water of Kathmandu Valley was below the permissible limit as recommended by WHO.^12^ A study in the Eastern Developmental region of Nepal on water samples of different sources. Few regions in their study were having fluoride levels above 0.10 mg/L and the majority of the area had fluoride levels below 0.05 mg/L as recommended by WHO.^13^ Another study conducted in Dharan also revealed similar findings in line with our study.^11^ Another study reported a low level of fluoride. Except for the Parsa district in their study settings which showed a fluoride level of 0.8 mg/L other study sites had a low level of Fluoride as recommended by WHO.^22^

The present study and those conducted earlier in Nepal have also revealed the spatial distribution of fluoride along the hilly region of Nepal and also confirms that the fluoride level in different geographical settings also varies.^14-16^ However, the findings have revealed the need for fluoridation and defluoridation schemes by a variety of means in Nepal. The study showed in Resunga Municipality there is a need for fluoridation schemes. The increased consumption of fluoridated water can be enhanced by community participation and school education. The increase in water fluoridation, milk fluoridation and salt fluoridation can be utilised in Resunga municipality.

Fluoride can be supplemented either in the form of systemic fluoride or topical fluoride. Of all the methods known, the fluoridation of water is the most economical fluoride delivery system.^21^ Fluoride can also be supplemented through drinking water and soft drinks, food, air, toothpaste, mouth gargles, fluoride supplements and tablets.^23^ In case of excess or deficiency of fluoride, water is the most effective mean for community water fluoridation in case of fluoride deficiency and start defluoridation in case of excess fluoride in water. Water fluoridation is normally a procedure carried out to put fluoride in water with either a dry feeder system or a saturator system.^24^ This method has also reduced dental caries by 5070%. Milk fluoridation was first done in Switzerland and is said to be effective only during early childhood. However, in the present day, in dental clinics, fluoride supplementation is also given via in-office methods.

Fluoride has been used excessively as a preventive measure in dentistry. Its use has been mostly focused on the reduction of dental caries, a condition that has affected many people throughout the world.^7^ The role of fluoride against dental caries is that it locally disrupts the carious mechanism.^5^ During the mineralization of teeth, fluoride acts as a favourable microelement for the oral cavity, however, it also has a role in the mineralization of bone.^25^ Community water fluoridation was initiated in the middle of the 20^th^ century as a means to prevent dental caries.^26^ The first water fluoridation was reported in the years 1945 and 1946 in the USA and Canada where a reduction of dental caries was observed.^27^ Achieving an optimum level of fluoride reduces the chances of dental caries and also slows down the progression of existing caries.^11^ Dental caries is a global public health problem. Current trends have shown the increase in fluoride consumption has led to a decrease in dental caries.^28^

In Nepal too, studies have shown an increased prevalence of dental caries. Subedi et al reported that caries status was higher in the age group of 5-6 years (69%) than in the age group of 12-13 years (53.23 %) in Kathmandu Valley.^29^ A study conducted in the Eastern part of Nepal also showed a 52% prevalence of dental caries in the 5-6 years age group,^30^ while in Western Nepal the caries prevalence was 67%.^31^

The study is not devoid of limitations. Even though the study findings could portray the fluoride status of Resunga Municipality, the researchers could collect only a few samples for a few wards. It would have another impact if the researchers could collect uniform samples from all the wards. This was due to the remote hills on these wards that were difficult to reach and these sites were the last ones to excel. This study is only based on the samples collected from Resunga Municipality so the results obtained do not imply the whole Gulmi district or Nepal. The result of the present study also points to the necessity of additional studies related to the prevalence of dental caries which can also act as a milestone for additional study.

## CONCLUSIONS

The study concluded that most of the water resources of Resunga municipality were below the optimum level of fluoride than that recommended by WHO. Prompt action is advised for policymakers for maintaining the water level of this region. The water fluoridation program has to be initiated while maintaining the optimum fluoride level recommended by WHO.

Other means of fluoride consumption, like the use of fluoridated toothpaste, has to be recommended. A prompt dental camp has to be conducted to observe caries' status too.

## References

[1] Mukherjee I, Singh UK, Patra PK (2019). Exploring a multi-exposure-pathway approach to assess human health risk associated with groundwater fluoride exposure in the semi-arid region of east India.. Chemosphere..

[2] Handa B (1975). Geochemistry and genesis of fluoride-containing ground waters in India.. Groundwater..

[3] Ozsvath DL (2009). Fluoride and environmental health: a review.. Reviews in Environmental Science and Bio/Technology..

[4] Hossain M, Patra PK (2020). Hydrogeochemical characterisation and health hazards of fluoride enriched groundwater in diverse aquifer types.. Environ Pollut..

[5] Cury J, Tenuta L (2008). How to maintain a cariostatic fluoride concentration in the oral environment.. Adv Dent Res..

[6] Fordyce F, Vrana K, Zhovinsky E, Povoroznuk V, Toth G, Hope B (2007). A health risk assessment for fluoride in Central Europe.. Environ Geochem Health..

[7] Hardwick K, Barmes D, Writer S, Richardson LM (2000). International collaborative research on fluoride.. J Dent Res..

[8] Purusothaman S, Dv L, Kumar RKL (2021). estimation of fluoride levels in drinking water and its association with acquired hypothyroidism in children - a prospective observational study.. International Journal of Current Research and Review..

[9] Li Y, Liang C, Slemenda CW, Ji R, Sun S, Cao J (2001). Effect of long-term exposure to fluoride in drinking water on risks of bone fractures.. J Bone Miner Res..

[10] Das G, Tirth V, Arora S, Algahtani A, Kafeel M, Alqarni AHG (2020). Effect of Fluoride Concentration in Drinking Water on Dental Fluorosis in Southwest Saudi Arabia.. Int J Environ Res Public Health..

[11] Koirala B, Niraula SR, Ghimire A (2020). Estimation of fluoride content of drinking water in Dharan.. Journal of BP Koirala Institute of Health Sciences..

[12] Shah P, Khanal S (2020). Estimation of fluoride concentration in drinking water of Kathmandu valley.. Nepal Med Coll J..

[13] Singh A, Shrestha A, Bhagat T (2018). Fluoride level in drinking water sources of Eastern Nepal.. J Nepal Health Res Counc..

[14] Akuno M, Nocella G, Milia E, Gutierrez L (2019). Factors influencing the relationship between fluoride in drinking water and dental fluorosis: a ten-year systematic review and meta-analysis.. J Water Health..

[15] Mamatha P, Rao SM (2010). Geochemistry of fluoride rich groundwater in Kolar and Tumkur Districts of Karnataka.. Environmental Earth Sciences..

[16] Webb E, Stewart C, Sami E, Kelsey S, Fairbairn Dunlop P, Dennison E (2021). Variability of naturally occurring fluoride in diverse community drinking-water sources, Tanna Island, Vanuatu.. Journal of Water, Sanitation and Hygiene for Development..

[17] Tatevossian A (1990). Fluoride in dental plaque and its effects.. J Dent Res..

[18] Castilho LSd, Velasquez LNM, Fantinel LM, Perini E (2010). Beliefs and attitudes about endemic dental fluorosis among adolescents in rural Brazil.. Rev Saude Publica..

[19] McDonagh MS, Whiting PF, Wilson PM, Sutton AJ, Chestnutt I, Cooper J (2000). Systematic review of water fluoridation.. BMJ..

[20] World Health Organization. (2011). Guidelines for drinking-water quality [Internet]..

[21] Walia T, Fanas SA, Akbar M, Eddin J, Adnan M (2017). Estimation of fluoride concentration in drinking water and common beverages in United Arab Emirates (UAE).. Saudi Dent J..

[22] Karki S, Humagain M, Seppänen M, Päkkila J, Anttonen V (2018). Oral health status associated with sociodemographic factors of Nepalese schoolchildren: a population-based study.. Int Dent J..

[23] Abouleish MYZ (2016). Evaluation of fluoride levels in bottled water and their contribution to health and teeth problems in the United Arab Emirates.. Saudi Dent J..

[24] Kabilan A, Ganapathy D, Jain AR (2018). Estimation of fluoride content in the drinking water in Chennai-A pilot study.. Drug Invention Today..

[25] Cerklewski FL (1997). Fluoride bioavailability-nutritional and clinical aspects.. Nutrition research..

[26] Parnell C, Whelton H, O'mullane D (2009). Water fluoridation.. Eur Arch Paediatr Dent..

[27] Browne D, Whelton H, O'Mullane D (2005). Fluoride metabolism and fluorosis.. J Dent..

[28] Whelton H, Spencer A, Do L, Rugg-Gunn A (2019). Fluoride revolution and dental caries: evolution of policies for global use.. J Dent Res..

[29] Subedi B, Shakya P, Kc U, Jnawali M, Paudyal B, Acharya A (2011). Prevalence of dental caries in 5-6 years and 12-13 years age group of school children of Kathmandu valley.. J Nepal Med Assoc..

[30] Koirala S, David J, Khadka R, Yee R (2003). Dental caries prevalence, experience and treatment needs of 5-6 year old, 12-13 year old and 15 year old schoolchildren of Sunsari district, Nepal.. Journal of the Nepal Dental Association..

[31] Yee R, McDonald N (2002). Caries experience of 5-6-year-old and 12-13-year-old schoolchildren in central and western Nepal.. International dental journal..

